# Engineering optical properties of gold-coated nanoporous anodic alumina for biosensing

**DOI:** 10.1186/1556-276X-9-414

**Published:** 2014-08-21

**Authors:** Laura P Hernández-Eguía, Josep Ferré-Borrull, Gerard Macias, Josep Pallarès, Lluís F Marsal

**Affiliations:** 1 Department of Electronic, Electric and Automatics Engineering, Universitat Rovira i Virgili, Avinguda dels Països Catalans 26, Tarragona 43007, Spain

**Keywords:** Nanoporous anodic alumina, Gold coating, Thin-film, Reflectance spectroscopy, Pore widening, Effective medium approximation, Modeling, Fabry-Pérot interferences

## Abstract

The effect in the Fabry-Pérot optical interferences of nanoporous anodic alumina films coated with gold is studied as a function of the porosity and of the gold thickness by means of reflectance spectroscopy. Samples with porosities between 14 and 70% and gold thicknesses (10 and 20 nm) were considered. The sputtering of gold on the nanoporous anodic alumina (NAA) films results in an increase of the fringe intensity of the oscillations in the spectra resulting from Fabry-Pérot interferences in the porous layer, with a reduction in the maximum reflectance in the UV-visible region. For the thicker gold layer, sharp valleys appear in the near-infrared (IR) range that can be useful for accurate spectral shift measurements in optical biosensing. A theoretical model for the optical behavior has also been proposed. The model shows a very good agreement with the experimental measurements, what makes it useful for design and optimization of devices based on this material. This material capability is enormous for using it as an accurate and sensitive optical sensor, since gold owns a well-known surface chemistry with certain molecules, most of them biomolecules.

## Background

Nanoporous anodic alumina (NAA) is one of the smartest materials in which scientists have centered their research with considerable interest in recent years [1,2] due to their physicochemical properties like thermal stability, environmental toughness, and biocompatibility. Alumina has been studied for decades [3]. The fabrication technology permits to obtain highly ordered and customized porous nanostructures that makes NAA very attractive for different applications such as nanomaterial synthesis [4,5], photonics [6], or sensors [7-9].

In particular, NAA has demonstrated its sensing capabilities: a great wealth of work has been carried out with this material in biotechnology areas [10], and it presents reliable possibilities of working as portable chemical and biochemical sensors [11], as well as label-free biosensors [12]. Furthermore, if the optical waveguide properties of NAA are exploited, much higher sensitivities than conventional surface plasmon resonance (SPR) sensors [2,13,14] can be achieved. Sensors based on alumina improve their sensitivity by the measurement of the oscillations in the reflectance spectrum produced by the Fabry-Pérot (F-P) interferences in a NAA thin film [15,16]. More specifically, sensors based on reflection interference spectroscopy (RIfS) have been developed with favorable results [17,18]. The variation of these oscillations upon analyte detection is the sensing principle. It is well known that the fringe intensity (FI) of the F-P interference pattern depends on the internal reflectivity of the mirrors composing the F-P cavity [19]. A F-P interferometer consists essentially of two plates with parallel reflecting plane surfaces (with some small transmittivity). When illuminated at near-normal incidence, a multiple-beam interference is generated that results in the maxima and minima in the reflectance or transmittance spectra.

In this work, a technique to improve the FI and consequently the sensitivity of NAA-based sensors is studied, and a model to predict the optical response and evaluate the material sensitivity has been developed. For this purpose, the UV-visible-infrared (IR) spectra of different NAA thin films obtained with different pore diameters (*D*_p_) were investigated before and after the deposition of a thin gold layer on its surface. This optical characterization will allow determining the geometric properties of the porous alumina. The gold layer increases the reflection coefficient at the NAA-medium interface and improves the FI. The measured spectra were compared with numerical simulations in order to establish a model based on the effective medium approximation to account for the porous nature of the material [20] and to obtain a tool for the evaluation of the structure sensitivity.

## Methods

### NAA sample fabrication

The NAA samples were fabricated by the well-known two-step anodization process [21,22]. First, samples were cleaned employing deionized (DI) H_2_O, EtOH, and again DI H_2_O and electropolished in a mixture of EtOH and HClO_4_ 4:1 (*v*/*v*) at 20 V and 5°C for 4 min. During the electropolishing process, the stirring rotation was alternated from clockwise to counterclockwise every 60 s in order to avoid stripes in the samples due to the stirring direction. Immediately after, the first anodization step was carried out in an aqueous solution of H_2_C_2_O_4_ 0.3 M as electrolyte at 40 V and 5°C for 20 h in order to obtain 10% porosity for maximum self-ordering of pores [23]. The obtained alumina film in the first step was dissolved by wet chemical etching in a mixture of H_3_PO_4_ 0.4 M and chromic acid H_2_CrO_7_ 0.2 M at 70°C for 3 h 30 min. The second anodization step was performed under the same conditions as the previous one. Finally, the pore diameter was modulated by applying a wet chemical etching after the anodization procedure in an aqueous solution of H_3_PO_4_ 5 wt% for a given time *t*_PW_ of 0, 6, 12, and 18 min.

### Surface coating of NAA samples and thickness calibration

Gold was sputtered on the samples at 0.05 mbar and 30 mA during 21 or 45 s, to obtain 10- or 20-nm gold overlayers on the NAA, respectively, employing a sputter coater Bal-Tec SCD 004 (Bal-Tec, Balzers, Liechtenstein). X-ray reflectometry was used in advance to calibrate the sputtering process and estimate the average deposited thickness. These X-ray reflectometry measurements were made using a Bruker-AXS D8-Discover diffractometer (Bruker AXS, Inc., Madison, WI, USA) with parallel incident beam (Göbel mirror) and vertical theta-theta goniometer, XYZ motorized stage mounted on an Eulerian cradle, incident and diffracted-beam Soller slits, a 0.01° receiving slit, and a scintillation counter as a detector. The angular 2 T diffraction range was between 0.4 and 5°. The data were collected with an angular step of 0.004° at 10 s per step. Cu_kα_ radiation was obtained from a copper X-ray tube operated with variable voltage (kV) and current (mA).

### Structural and optical characterization of samples

The NAA samples were characterized by an environmental scanning electron microscope (ESEM; FEI Quanta 600, Hillsboro, OR, USA) and field emission SEM (Schottky FE) 4 pA to 20 nA, 0.1 to 30 kV and 1.1 nm. The specular reflectance measurements were performed in a PerkinElmer Lambda 950 UV/VIS/NIR spectrometer (PerkinElmer, Waltham, MA, USA) with a tungsten lamp used as excitation light source. The standard image-processing package (ImageJ, public domain program developed at the RSB of the NIH, USA) was used to carry out the SEM image analysis [24].

## Results and discussion

**Figure 1:**
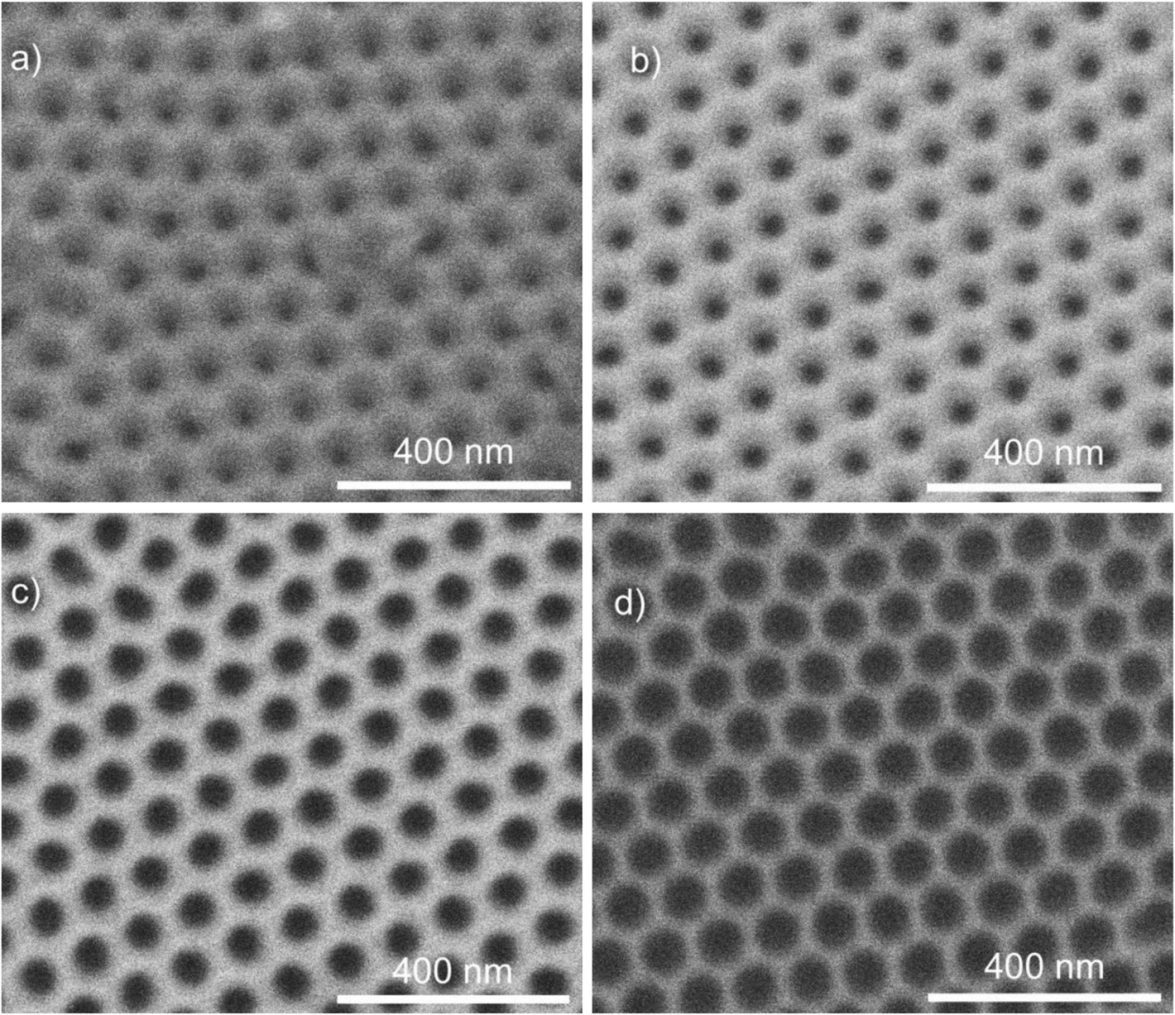
**SEM top view images of NAA samples with four different pore widening times (*t***_**PW**_**). (a)** As-produced, *t*_PW_ = 0 min; **(b)** *t*_PW_ = 6 min; **(c)** *t*_PW_ = 12 min; and **(d)** *t*_PW_ = 18 min.

Figure 1 shows four SEM top view images of four samples obtained after the different pore widening times. All the figures have the same scale in order to enable a comparison of pore sizes and interpore distances. In all cases, a good self-arrangement of the pores in a hexagonal lattice can be observed. The pore size increases as expected with the pore widening time. The average interpore distance estimated by means of image processing from these images is *D*_int_ = 102 nm. Image processing can also be used to approximately estimate the average pore diameter. Nevertheless, this estimation is approximate since the actual pore walls are not precisely defined in the pictures. This approximate estimation is detailed in Table 1.

**Table 1:** Results from the SEM image characterization of the samples after the pore widening and before the deposition of gold

**Pore widening time (min)**	**Estimated pore diameter, *D***_**p **_**(nm)**	**Standard deviation (nm)**
0	29	4
6	36	2
12	57	3
18	79	2

**Figure 2:**
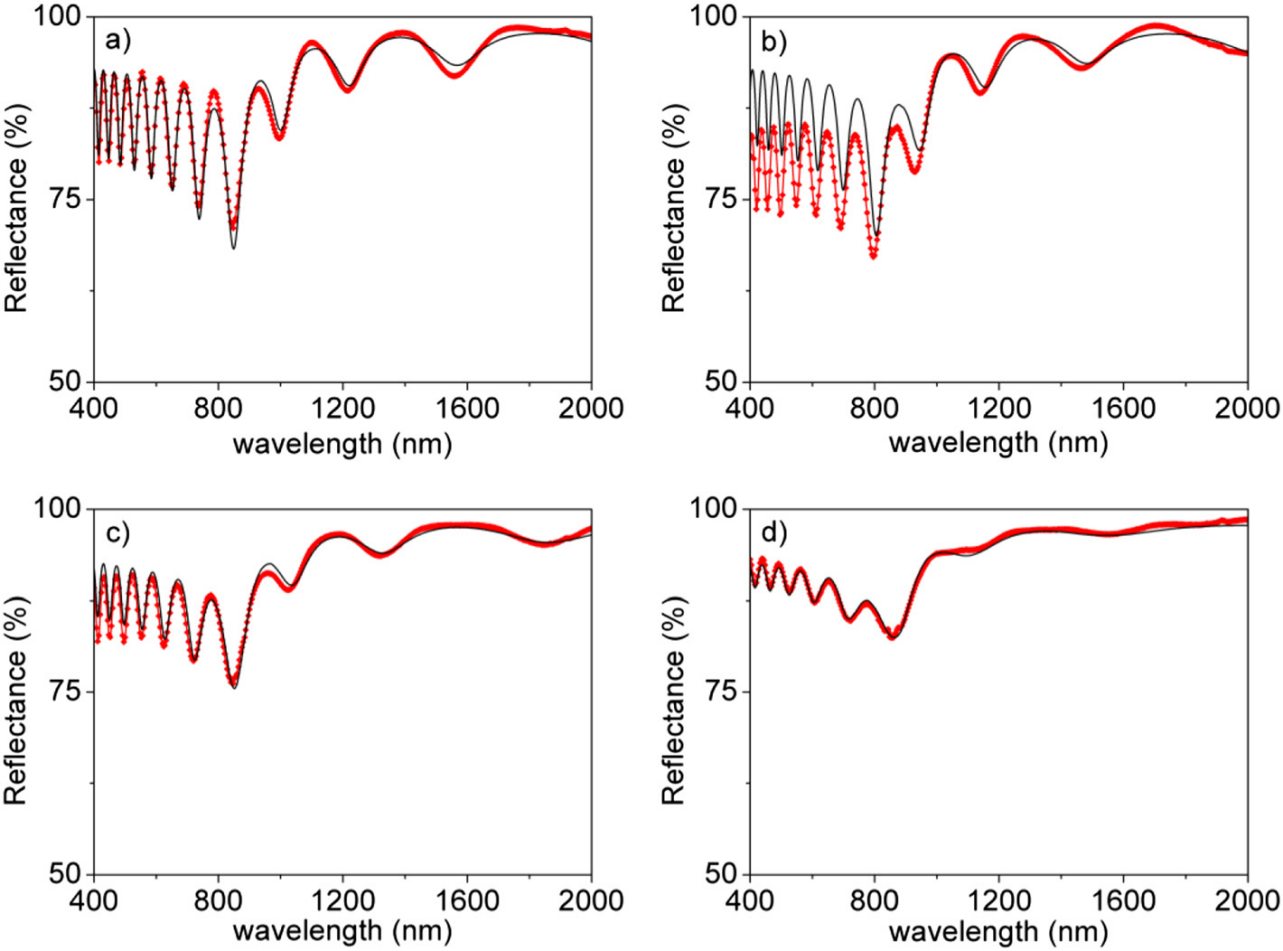
**Reflectance spectra of samples with different *t***_**PW **_**before gold deposition.** Red symbols joined with solid red line represent experimentally measured reflectance spectra. Solid black line represents the best least-square fit corresponding to simulation. **(a)** *t*_PW_ = 0 min, **(b)** *t*_PW_ = 6 min, **(c)** *t*_PW_ = 12 min, and **(d)** *t*_PW_ = 18 min.

The samples were first optically characterized in reflectance prior to the deposition of gold on the top surface. The measured reflectance spectra are shown in Figure 2 (red dots joined with red solid lines) for the four *t*_PW_. The spectra present oscillations generated by Fabry-Pérot interferences in the optical cavity constituted by the NAA film surrounded by the incident medium (air) and the substrate (aluminum). The frequency and amplitude (fringe intensity (FI)) of the reflectance oscillations can be analyzed to obtain important information about the geometry of the NAA films. The number of oscillations depends on the effective optical thickness (EOT) of the NAA layer, which is directly related with the refractive index of the NAA layer. On the other hand, the FI depends on the refractive index contrast between the NAA layer and the surrounding materials (the substrate and the incident medium in this case). Both the number of oscillations and their FI decrease with increasing *t*_PW_, what indicates the consequent decrease in the NAA film effective refractive index.

This analysis is performed more systematically by means of a fitting to a theoretical model of the sample. The same Figure 2 shows one calculated reflectance spectrum for each *t*_PW_. These spectra are calculated assuming an optical model for the samples consisting of (i) the substrate (aluminum), (ii) the NAA porous layer, and (iii) the incident medium (air). The porous layer, in turn, is considered as a mixture of aluminum oxide and air, with thickness *d* = 1,620 nm. The Bruggeman effective medium approximation is used to obtain the refractive index of the porous layer (*n*_eff_) from the refractive index of the aluminum oxide [25] and that of air (*n*_air_ = 1) taking into account the corresponding volume fractions:

(1) $$\left(1-P\right)\frac{n_{\mathrm{Alumina}}^{2}-n_{\mathrm{eff}}^{2}}{n_{\mathrm{Alumina}}^{2}+2n_{\mathrm{eff}}^{2}}+P\frac{1-n_{\mathrm{eff}}^{2}}{1+2n_{\mathrm{ef}f}^{2}}=0.$$

These volume fractions are related to the porosity *P* of the porous layer, *P* being the volume fraction of air and 1 - *P* the volume fraction of aluminum oxide. The calculated reflectance spectra shown in Figure 2 correspond to the best least-square fit obtained by varying the porosity of the layer.

Table 2 summarizes the obtained results for the four *t*_PW_. Besides the porosity that gives the best fit of the model with the experimental measurements, Table 2 also reports the corresponding effective refractive index at 660 nm and the estimated pore diameter (*D*_p_) obtained from the porosity and the interpore distance *D*_int_ (previously estimated from the SEM pictures). Assuming a perfect hexagonal arrangement, these magnitudes are related through the following expression:

**Table 2:** Results from the optical characterization of the samples after the pore widening and before the deposition of gold

**Pore widening time (min)**	**NAA film porosity, *P *** **(%)**	**NAA film effective refractive index, *n***_**eff**_	**Estimated pore diameter, *D***_**p **_**(nm)**
0	14.3	1.65	38.6
6	23.1	1.58	51.2
12	44.6	1.41	72.3
18	71.2	1.20	90.9

(2) $$D_{p}=D_{\text{int}}\cdot \sqrt{\frac{P\cdot 2\cdot \sqrt{3}}{\Pi }}\cdot$$

Comparing the *D*_p_ obtained from this optical characterization method with the approximate estimation from SEM, it can be seen that both show an increasing trend but that pore size determinations are not very precise from image analysis of surface pictures.

**Figure 3:**
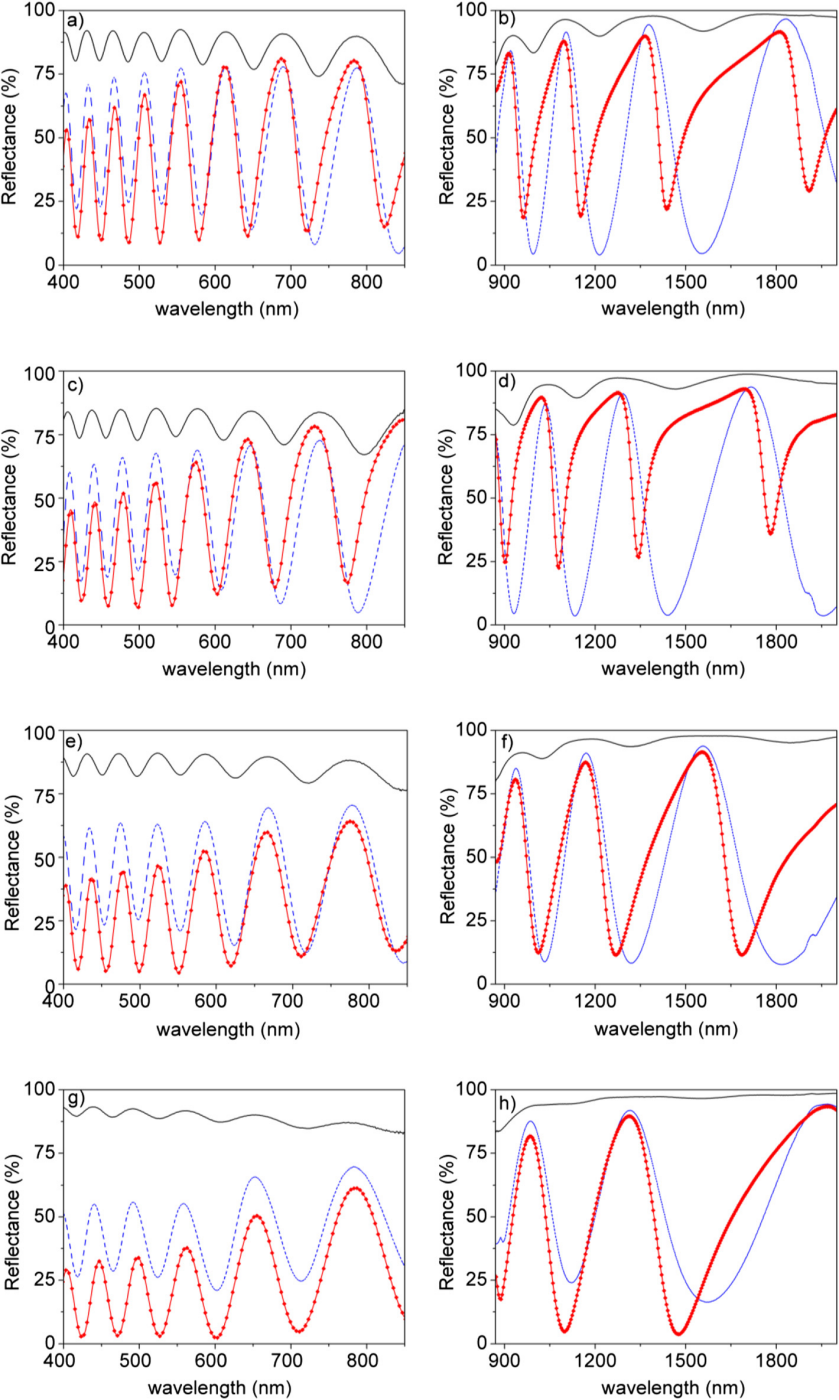
**Reflectance spectra of samples with different *t***_**PW **_**before gold deposition and after sputtering 10- and 20-nm gold on NAA.** Solid black line represents samples without gold. Dashed blue line represents samples with 10 nm sputtered gold. Red symbols joined with red lines represent samples with 20 nm of gold. Plots on the left correspond to the UV–vis spectral region, while plots on the right correspond to the near-IR spectral region. **(a, b)** *t*_PW_ = 0 min, **(c, d)** *t*_PW_ = 6 min, **(e, f)** *t*_PW_ = 12 min, and **(g, h)** *t*_PW_ = 18 min.

Figure 3 shows the reflectance spectra for the four *t*_PW_ after the sputtering of 10 nm (dashed blue line) and 20 nm (red dots joined with red solid lines) of gold on top of the NAA films. The spectra for the same samples before gold deposition are also shown for comparison purposes. The spectra are divided in the UV-visible region (left) and in the near-IR region (right) to improve the visibility of the oscillations, as their frequency is higher in the UV-visible region. With the deposition of gold, the FI of the samples increases significantly while the number of oscillations remains constant and only a small blue shift of the oscillations can be realized. The increase in FI is due to the increase in refractive index contrast between the NAA film and the deposited gold layer. However, for increasing NAA film porosity, the FI of the gold-coated samples decreases in the same way as it happened for the as-produced samples. Another remarkable feature of the spectra in the UV-visible range is that the maximum measured reflectance decreases for increasing *t*_PW_. In this region, gold has its stronger absorption at 500 nm, making the reflectivity of light decrease [26]. This decrease is stronger for the samples with 20 nm of deposited gold.

In the near-IR range, the spectra show bigger differences: the reflectance for the samples with 10 nm of gold show symmetric oscillations with respect to the reflectance minima, while for 20 nm of gold, the oscillations are asymmetric. Furthermore, the position of the minima is clearly blue shifted in the samples with 20 nm of gold with respect to the samples without and with 10 nm of gold. It is important to remark that this asymmetry and blue shift decrease with increasing *t*_PW_ and that for the two lower porosities (corresponding to *t*_PW_ = 0 min and *t*_PW_ = 6 min), this asymmetry results in narrow valleys with small width and a well-defined minimum wavelength that can be useful in the detection of spectral shifts.

If the FI between the samples with 10 and 20 nm of deposited gold is compared, it can be concluded that the relation of the FI with the gold thickness is strongly dependent on the porosity of the NAA film: for the lower porosities, the FI for the 10 nm gold-coated samples is bigger, but this trend is reversed as the porosity increases.

**Figure 4:**
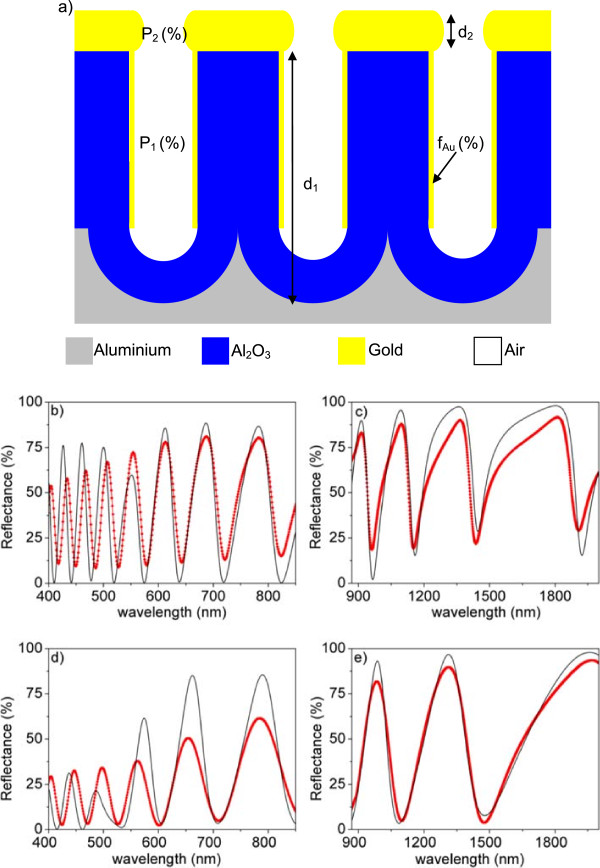
**Model for cold-coated NAA samples and comparison of the measured and the best least-squares fitting simulated reflectance spectra. (a)** Schematic drawing of the proposed theoretical model for gold-coated NAA samples. Red symbols joined with solid red line represent experimentally measured reflectance spectra. Solid black line represents best least-square fit corresponding to simulation. Plots on the left correspond to the UV–vis spectral region, while plots on the right correspond to the near-IR spectral region. **(b, c)** *t*_PW_ = 0 min and **(d, e)** *t*_PW_ = 18 min.

In this work, we aim at proposing a model that can explain all the behaviors observed in Figure 3 and specially the dependence of the spectra with the porosity and the deposited gold thickness in the near-IR range, where the structure might be more useful in the detection of wavelength changes because of the existence of the narrow valley characteristics. The model we propose here is composed of two thin layers on the aluminum substrate, as depicted in Figure 4a. The first layer, in contact with the aluminum substrate, corresponds to the NAA film (equivalent to the NAA film used in the model considered to obtain the fits in Figure 2) but with a small amount of gold deposited on the inner pore walls, to take into account that a certain amount of gold can infiltrate the pores in the sputtering process. This first layer is characterized by its thickness (*d*_1_), the porosity (*P*_1_), and the volume fraction of gold in the effective medium (*f*_Au_). The second layer consists of a porous gold film corresponding to the sputtered gold layer on the NAA. This gold porous film is characterized by its thickness (*d*_2_) and its porosity (*P*_2_). Figure 4b, c shows the best fits obtained with this model for *t*_PW_ = 0 min, while Figure 4d, e corresponds to *t*_PW_ = 18 min, both cases for the samples with 20 nm of sputtered gold. The experimental data are represented as dots joined with lines while the best least-square fits obtained using the model are represented as a solid line. The parameter values corresponding to this best fit are specified in Table 3.

**Table 3:** **Results from the optical characterization of the samples with *t***_**PW**_ **= 0 min and *t***_**PW**_ **= 18 min after the deposition of 20 nm of gold**

**Pore widening time (min)**	**NAA film porosity, *P***_**1 **_**(%)**	**Volume fraction of gold in the NAA film, *f***_**Au **_**(%)**	**NAA film thickness, *d***_**1 **_**(nm)**	**Gold film porosity, *P***_**2 **_**(%)**	**Gold film thickness, *d***_**2 **_**(nm)**
0	6,8	0.1	1,580	55.3	30
18	69.3	1.2	1,580	59.5	25

The model is able to explain the reduction of the reflectance maxima in the UV-visible range by the small amount of gold that can penetrate into the pores (0.1% for *t*_PW_ = 0 min and 1.2% for *t*_PW_ = 18 min). These results are consistent with the pore size, as a bigger amount of gold can penetrate for bigger pores. Nevertheless, the model predicts a smaller reflectance reduction than what is observed in the measurements. This is due to the fact that there possibly exist other sources of loss in this spectral range than the absorption from the gold in the inner pore walls. Such losses can arise from scattering or plasmonic effects that the model cannot take into account. In the near-IR range, the changes in the shape of the oscillations are explained by the differences in thickness and porosity of the gold layer on the NAA film. The obtained gold film porosities are also consistent with the porosity of the NAA film (*P*_2_ = 55.3% for *t*_PW_ = 0 min and *P*_2_ = 59.5% for *t*_PW_ = 18 min), bigger for the bigger NAA film porosity. This result is in good agreement with previous works [27] where a 10-nm-thickness gold layer is sputtered onto NAA. Cross-sectional FE-SEM pictures in this work show that sputtered gold does not penetrate into the NAA pores and forms a superficial film. With just these two parameters (thickness and porosity), it is possible to account for all the features observed in the spectra in the near-IR range: the narrow asymmetric valleys for the low-porosity NAA that become more symmetric as the porosity increases and the differences in blue shift of the reflectance minima.

## Conclusions

In this work, we have shown the effect on the reflectance spectra of nanoporous anodic alumina films of the sputtering of a gold overlayer, as a function of the NAA porosity and of the gold thickness. The results show that the gold overlayer improves dramatically the contrast of the oscillations in the reflectance spectrum, what would result in an improvement of NAA-based optical sensors. By adequately tuning the gold thickness, sharp valleys in the reflectance can be obtained in the near-IR range that can further contribute to a more accurate determination of spectral shifts and a consequent sensitivity improvement. A model based on the effective medium approximation for the NAA layer and for the deposited gold thin film has been proposed and shows a good agreement with the experimental measurements. In particular, the model is able to explain the shape of the sharp reflectance valleys in the near-IR for the different gold thicknesses and NAA porosities.

This work shows that nanoporous anodic alumina coated with gold is a promising structure for future biosensing applications because of the improved sensitivity in any pore geometry due to the enhancement in the reflectance FI. Specific applications could then benefit from a big surface-to-volume ratio in big porosity structures to sense biomolecules, whereas for filtering purposes, the pore diameter can be tuned to match the molecule size to be transported through the membrane.

## Abbreviations

NAA: nanoporous anodic alumina; FI: fringe intensity; SPR: surface plasmon resonance; F-P: Fabry-Pérot; RIfS: reflection interference spectroscopy; EOT: effective optical thickness; *n*_eff_: porous layer effective refractive index; *D*_int_: interpore distance; *D*_p_: pore diameter; *t*_PW_: pore widening time; P: film porosity.

## Competing interests

The authors declare that they have no competing interests.

## Authors’ contributions

LPHE designed the geometry of the NAA samples, carried out the reflectance measurements, performed the simulations, and drafted the manuscript. GM fabricated the NAA samples and helped in the manuscript elaboration. JFB made substantial contributions to the analysis and interpretation of theoretical simulations, and JP and LFM coordinated all the experiments and gave final approval of the version to be submitted. All authors help to draft the article and approved the final manuscript.

## References

[B1] Losic D Simovic S Self-ordered nanopore and nanotube platforms for drug delivery applications Expert Opin Drug Deliv 2009 9 1363 1381 10.1517/17425240903300857 19860534

[B2] Yeom S-H Kim O-G Kang B-H Kim K-J Yuan H Kwon D-H Kim H-R Kang S-W Highly sensitive nano-porous lattice biosensor based on localized surface plasmon resonance and interference Opt Express 2011 9 22882 22891 10.1364/OE.19.022882 22109166

[B3] Thompson GE Wood GC Porous anodic film formation on aluminium Nature 1981 9 230 232 10.1038/290230a0

[B4] Shingubara S Fabrication of nanomaterials using porous alumina templates J Nanopart Res 2003 9 17 30

[B5] Zhang Z Shimizu T Senz S Gösele U Ordered high-density Si [100] nanowire arrays epitaxially grown by bottom imprint method Adv Mater 2009 9 2824 2828 10.1002/adma.200802156

[B6] Maksymov I Ferré-Borrull J Pallarès J Marsal LF Photonic stop bands in quasi-random nanoporous anodic alumina structures Photon Nanostruct Fundam Appl 2012 doi:10.1016/j. photonics.2012.02.003

[B7] Kim D-K Kerman K Yamamura S Kwon YS Takamura Y Tamiya E Label-free optical detection of protein antibody-antigen interaction on Au capped porous anodic alumina layer chip Jpn J Appl Phys 2008 9 1351 1354 10.1143/JJAP.47.1351

[B8] Koutsioubas AG Spiliopoulos N Anastassopoulos D Vradis AA Priftis GD Nanoporous alumina enhanced surface plasmon resonance sensors J Appl Phys 2008 9 094521 10.1063/1.2924436

[B9] Varghese OK Gong D Dreschel WR Ong KG Grimes CA Ammonia detection using nanoporous alumina resistive and surface acoustic wave sensors Sens Act B 2003 9 27 35 10.1016/S0925-4005(03)00252-1

[B10] Ingham CJ ter Maat J de Vos WM Where bio meets nano: the many uses for nanoporous aluminum oxide in biotechnology Biotechnol Adv 2012 9 1089 1099 10.1016/j.biotechadv.2011.08.005 21856400

[B11] Santos A Kumeria T Losic D Nanoporous anodic aluminum oxide for chemical sensing and biosensors TrAC Trends Anal Chem 2013 9 25 38

[B12] Hotta K Yamaguchi A Teramae N Deposition of polyelectrolyte multilayer film on a nanoporous alumina membrane for stable label-free optical biosensing J Phys Chem C 2012 9 23533 23539 10.1021/jp308724m

[B13] Hotta K Yamaguchi A Teramae N Nanoporous waveguide sensor with optimized nanoarchitectures for highly sensitive label-free biosensing ACS Nano 2012 9 1541 1547 10.1021/nn204494z 22233297

[B14] Lau KHA Tan L-S Tamada K Sander MS Knoll W Highly sensitive detection of processes occurring inside nanoporous anodic alumina templates: a waveguide optical study J Phys Chem B 2004 9 10812 10818 10.1021/jp0498567

[B15] Huang K Pu L Shi Y Han P Zhang R Zheng YD Photoluminescence oscillations in porous alumina films Appl Phys Lett 2006 9 201118 10.1063/1.2390645

[B16] Lin VSY Motesharei K Dancil KPS Sailor MJ Ghadiri MR A porous silicon-based optical interferometric biosensor Science 1997 9 840 843 10.1126/science.278.5339.840 9346478

[B17] Santos A Balderrama VS Alba M Formentín P Ferré-Borrull J Pallarès J Marsal LF Tunable Fabry-Pérot interferometer based on nanoporous anodic alumina for optical biosensing purposes Nanoscale Res Lett 2012 9 370 10.1186/1556-276X-7-370 22759928 PMC3413587

[B18] Kumeria T Losic D Controlling interferometric properties of nanoporous anodic aluminium oxide Nanoscale Res Lett 2012 9 88 10.1186/1556-276X-7-88 22280884 PMC3287969

[B19] Born M Wolf E Principles of Optics 1986 6 corrth Oxford: Pergamon Press 329

[B20] Bosch S Ferré-Borrull J Sancho-Parramon J A general-purpose software for optical characterization of thin films: specific features for microelectronic applications Solid State Electron 2001 9 703 709 10.1016/S0038-1101(01)00092-2

[B21] Masuda H Fukuda K Ordered metal nanohole arrays made by a two-step replication of honeycomb structures of anodic alumina Science 1995 9 14661468 10.1126/science.268.5216.1466 17843666

[B22] Santos A Balderrama VS Alba M Formentín P Ferré-Borrull J Pallarès J Marsal LF Nanoporous anodic alumina barcodes: toward smart optical biosensors Adv Mater 2012 9 1050 1054 10.1002/adma.201104490 22266815

[B23] Nielsch K Choi J Schwirn K Wehrspohn RB Gösele U Self-ordering regimes of porous alumina: the 10% porosity rule Nano Lett 2002 9 677 680 10.1021/nl025537k

[B24] Abràmoff MD Magalhaes PJ Ram SJ Image processing with ImageJ Biophoton Int 2004 9 36 42

[B25] Palik ED Handbook of Optical Constants of Solids 1998 San Diego, CA: Academic

[B26] Ahmad N Stokes J Fox NA Teng M Cryan MJ Ultra-thin metal films for enhanced solar absorption Nano Energy 2012 9 777 782 arXiv: 1202.6603V2 [physics.optics] 10.1016/j.nanoen.2012.08.004

[B27] Macías G Hernández-Eguía LP Ferré-Borrull J Pallares J Marsal LF Gold-coated ordered nanoporous anodic alumina bilayers for future label-free interferometric biosensors ACS Appl Mater Interf 2013 9 8093 8098 10.1021/am4020814 23910449

